# A case of esophageal carcinoma due to esophageal squamous papillomatosis

**DOI:** 10.1016/j.ijscr.2020.02.057

**Published:** 2020-02-29

**Authors:** E. Fraga, J. Almeida, C. Camacho, J. Simões, A. Bernardes

**Affiliations:** a General Surgery Department, Coimbra Hospital and University Center, Coimbra, Portugal; b Faculty of Medicine, University of Coimbra, Coimbra, Portugal

**Keywords:** ESP, Esophageal squamous papilloma, SPE, squamous papillomatosis of the esophagus, EGD, esophagogastroduodenoscopy, GERD, Gastroesophageal reflux disorder, HPV, human papillomavirus, Ivor-Lewis esophagectomy, Esophagogastroduodenoscopies, Esophageal papillomatosis, Esophageal papilloma, Squamous cell carcinoma

## Abstract

**Introduction:**

Squamous Papilloma is a rare benign tumor of the esophagus. Esophageal squamous papilloma is thought to arise from a chronic inflammatory reaction due to mechanical or chemical irritant. ESP is usually considered to have a benign clinical course; however, some reports highlight the potential development of a malignancy. The development of extensive esophageal squamous papillomas also known as squamous papillomatosis of the esophagus is even less frequent.

**Presentation of the case:**

We present the case of an esophageal papillomatosis that developed into an invasive esophageal squamous cell carcinoma that was only diagnosed in the surgical specimen after minimally invasive Ivor-Lewis esophagectomy and in this case report, we discuss its etiology, diagnosis and treatment.

**Conclusion:**

Extensive papillomatosis with continuous symptoms, especially persistent dysphagia, should always prompt a thorough analysis into a possible underlying malignancy.

## Highlights

• Squamous Papilloma is a rare benign tumor of the esophagus.
• Esophageal squamous papilloma (ESP) is thought to arise from a chronic inflammatory reaction due to mechanical or chemical irritant.
• Esophageal squamous papilloma is considered to have a benign course; however, some reports highlight the potential development of a malignancy.
• Extensive papillomatosis with continuous symptoms (especially unremitting dysphagia and hematemesis) should always prompt a thorough investigation into a possible underlying malignancy.

## 1 Introduction

Esophageal squamous papillomas (ESPs) are rare epithelial tumors thought to be benign, most commonly appearing in 0.01–0.45% of all esophagogastroduodenoscopies (EGD) [1,[3], [4], [5], [6]]. They are usually asymptomatic, and so most lesions are found incidentally. ESPs may present in a multitude of clinical manifestations such as symptomatic dysphagia, anemia and hematemesis. The development of extensive esophageal squamous papillomas also known as squamous papillomatosis of the esophagus (SPE) is even less frequent with only a few cases being described in the literature.

We describe the case of an esophageal papillomatosis complicated by the development of an invasive esophageal squamous cell carcinoma, that was only diagnosed in the surgical specimen after minimally invasive Ivor-Lewis esophagectomy, despite multiple nondiagnostic biopsies.

This case has been reported in line with the SCARE criteria [12].

## 2 Case report

A 67-year-old Caucasian man visited his primary care physician with complaints of hematemesis and continuous dysphagia. He had history of cigarette smoking and social alcohol consumption. Previous medical history included hypertension and hyperlipidemia. An initial EGD showed multiple verrucous lesions extending from 30 cm to 39 cm from his incisors. Pathological examination of the multiple biopsies showed squamous papillomatosis.

The patient was referred for evaluation at our hospital and a new EGD showed the same extensive verrucous lesions with three large growths on the distal esophagus causing significant stenosis. He repeated the biopsies and maintained the previous histological diagnosis. A thoracoabdominal CT-Scan revealed large esophageal lesions apparently superficial with thickening of the esophageal wall. Endoscopic Ultrasound showed that these changes were almost all limited to the mucosa and submucosa except in one area where they appeared to penetrate deeper.

**Fig. 1:**
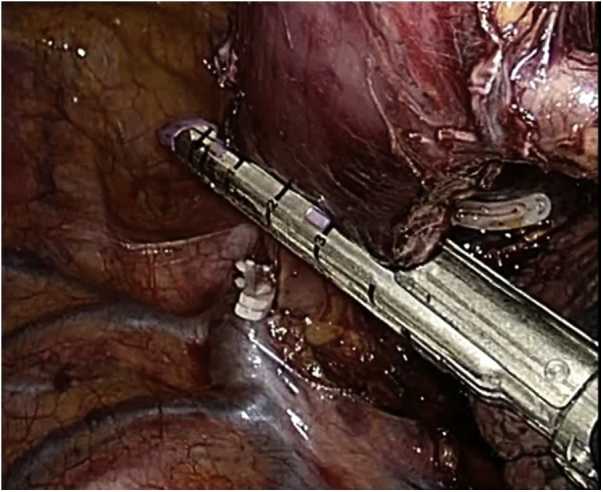
Thoracoscopic approach: esophageal resection below the azygos vein arch.

**Fig. 2:**
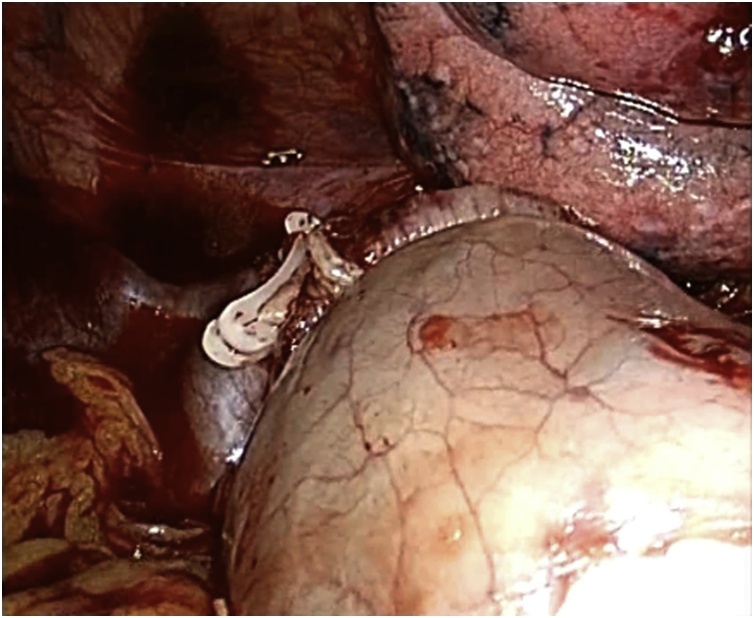
Thoracoscopic approach: esophago-gastric termino-lateral mechanical anastomosis.

At this time, the patient’s dysphagia got worse and there was substantial suspicion of potential malignancy. After a multidisciplinary discussion, he was proposed for surgery. He underwent a minimally invasive Ivor-Lewis Esophagectomy (laparoscopy + right lateral thoracoscopy) with a mechanical termino-lateral esophago-gastric upper thoracic anastomosis (at approximately 24/25 cm from the incisors) (Figs. 1, 2). The postoperative (PO) period was uneventful and the patient was discharged at the 9^th^ PO day.

**Fig. 3:**
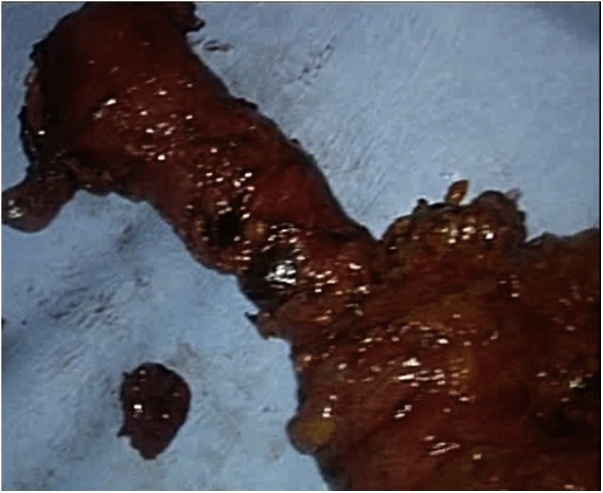
Esophagectomy specimen.

The examination of esophagectomy specimen (Fig. 3) showed a squamous cell carcinoma *in situ* (verrucous type) of the distal esophagus with areas of high dysplasia and extensive diffuse squamous papillomatosis. There were no signs of lymphatic or perineural invasion and margins were clear. The eighteen lymph nodes excised were all negative for malignancy. The TNM stage (8th edition) was Tis N0. One year later, at the follow-up consultation, there were no signs of recurrence.

## 3 Discussion

Esophageal squamous papillomas (ESPs) are epithelial tumors thought to be benign. These tumors are rare, occurring in about 0.01% of individuals at autopsy and 0.07% in an endoscopic series [2,5,6]. Studies indicate that ESP seems to be more common in middle aged individuals and show male dominance or nearly equal sex distribution in the western countries, but in the Asian countries, women are more commonly diagnose with ESP than men [3]. ESPs prevalence is increasing with some studies showing an increase from 0.13% to 0.57% per year from 2000 to 2013 and this may be related with the rise of HPV infection [4].

The pathogenesis of the esophageal papillomas is thought to arise from chronic inflammatory reaction due to a mechanical or chemical irritant such as gastroesophageal reflux disorder (GERD), alcohol, smoking, caustic injury, food impaction or human papillomatosis (HPV) infection or a combination of these factors [[1], [2], [3],^5^,^6^]. Pantham et al. described that 47.4% of papilloma lesions were associated with the high-risk HPV serotype 16 [4]. Tiftikçi et al. reported that the presence of high-risk type HPV serotype 16,18,31,81 may contribute to the pathogenesis of ESPs [10]. Bohn et al. also reported that 85.7% of papilloma lesions were associated with low-risk HPV serotypes [11].

ESPs are usually asymptomatic, and most lesions are found incidentally, but may present in a multitude of clinical manifestations such as symptomatic dysphagia, anemia and hematemesis [1]. About two-thirds of ESPs are found in the lower third of the esophagus althought they can also be located elsewhere [2,5]. When these lesions occur in the proximal third, they can suggest HPV infection. They usually appear as a single lesion with sizes averaging between 2–8 mm, so the development of extensive esophageal squamous papillomas also known as squamous papillomatosis of the esophagus (SPE) is uncommon and even less frequent [1,2]. Whenever possible, endoscopic removal is recommended [1,3]. Endoscopists must be aware of these lesions and remove them, because of their differential diagnosis. A repeat EGD is advised at 18–48 post-resection to assess for recurrence [5]. Currently, there are no guidelines for surveillance of ESPs [4].

While most ESPs seem to be benign, there is still some controversy about its malignant potential. Furthermore, in other areas of the body, papillomas are known to be precursors to squamous cell carcinoma, and as such there are concerns that ESP are premalignant lesions [1,5]. The latest reports show the potential progression to malignancy in association with ESP/SPE, also highlighting the difficulty in identifying the malignant areas within these lesions on routine endoscopy and biopsies, especially in SPE [[6], [7], [8], [9]]. Essentially, more information is necessary, as to better recognize their potential for malignant transformation and allow for better surveillance after removal or surgery [1]. Only a few cases of squamous papillomatosis of the esophagus that progressed to invasive squamous cell carcinoma have been described, thus highlighting the importance of this case report. In our case, endoscopic removal was not feasible due to the size and extent of the SPE. This case supports the recent literature indicating that ESP/SPE are not always benign, while showcasing the difficulties found, requiring surgery for a definite diagnosis and treatment.

The finding of extensive esophageal papillomatosis with evolving and continuous dysphagia or hematemesis should prompt a very thorough investigation regarding underlying associated malignancy.

## 4 Conclusion

An esophageal squamous cell carcinoma arising from squamous papillomatosis is a rare condition and may be difficult to diagnose.

Extensive papillomatosis with continuous symptoms (especially unremitting dysphagia and hematemesis) should always prompt a thorough investigation into a possible underlying malignancy and in some cases, only surgery will allow for a correct diagnosis and treatment. Overall, more information is needed on the long-term follow-up, as to better recognize their potential for malignant transformation and allow for better surveillance after removal or surgery.

Written informed consent was obtained from the patient for publication of this case report and accompanying images. A copy of the written consent is available for review by the Editor-in-Chief of this journal on request.

## Funding

No sponsors or funding.

## Ethical approval

This case report is exempt from ethnical approval in my institution.

## Consent

Written informed consent was obtained from the patient for publication of this case report and accompanying images. A copy of the written consent is available for review by the Editor-in-Chief of this journal on request.

## Author contribution

Emília Fraga – Concept and design, data curation and collection, writing the paper

João Almeida – Concept, Supervision and Writing - review

Cristina Camacho – Data collection and others

João Simões – Data collection and others

António Bernardes – Supervision and review

## Registration of research studies

The article is a case report.

## Guarantor

The article is a case report.

## Provenance and peer review

Not commissioned, externally peer-reviewed.

## Declaration of Competing Interest

All authors have no conflicts of interest.
